# Staphylococcal species less frequently isolated from human clinical specimens - are they a threat for hospital patients?

**DOI:** 10.1186/s12879-020-4841-2

**Published:** 2020-02-11

**Authors:** Magdalena Szemraj, Magdalena Grazul, Ewa Balcerczak, Eligia M. Szewczyk

**Affiliations:** 1 Department of Pharmaceutical Microbiology and Microbiological Diagnostic, Medical University of Lodz, Pomorska 137, 90-235 Łódź, Poland; 2 Department of Pharmaceutical Biochemistry and Molecular Diagnostic, Laboratory of Molecular Diagnostic and Pharmacogenomics, Medical University of Lodz, Łódź, Poland

**Keywords:** CoNS virulence, *Staphylococcus hominis*, Genes transfer

## Abstract

**Background:**

Coagulase-negative staphylococci belonging to *S. haemolyticus*, *S. hominis* subsp. *hominis*, *S. simulans,* and *S. warneri* are often described as etiological factors of infections. Staphylococci are a phylogenetically coherent group; nevertheless, there are differences among the species which may be important to clinicians.

**Methods:**

We investigated selected virulence factors and antibiotic resistance that were phenotypically demonstrated, the presence and expression of genes encoding the virulence factors, and the type of the SCCmec cassette.

**Results:**

The differences between the tested species were revealed. A great number of isolates produced a biofilm and many of them contained single *icaADBC* operon genes. Clear differences between species in the lipolytic activity spectrum could be related to their ability to cause various types of infections. Our studies also revealed the presence of genes encoding virulence factors homologous to *S. aureus* in the analysed species such as enterotoxin and *pvl* genes, which were also expressed in single isolates of *S. simulans* and *S. warneri*. *S. haemolyticus* and *S. hominis* subsp. *hominis* isolates were resistant to all clinically important antibiotics including ß-lactams. The identified SCC*mec* cassettes belonged to IV, V, VII, and IX type but most of the detected cassettes were non-typeable. Among the investigated species, *S. hominis* subsp. *hominis* isolates accumulated virulence genes typical for *S. aureus* in the most efficient way and were widely resistant to antibiotics.

**Conclusions:**

Our results clearly indicated significant differences between the tested species, which might be a result of the horizontal gene transfer (HGT) and can lead to the formation and selection of multi-drug resistant strains as well as strains with new virulence features. Such strains can have a new clinical relevance.

## Background

The clinical relevance of coagulase-negative staphylococci (CoNS) belonging to *Staphylococcus haemolyticus*, *S. hominis*, *S. simulans,* and *S. warneri* species is increasing*.* The appearance of automated identification systems improved their detection. Although staphylococci constitute a phylogenetically coherent group there are differences among the species important to clinicians [1].

According to the literature, *S. haemolyticus* is the second major species among CoNS responsible for health care associated infections [2, 3]. It causes blood infections, sepsis, and is often isolated from ocular infections [4]. It is also detected as a cause of otitis, peritonitis, and urinary tract infections [5]. It is noteworthy to mention that *S. haemolyticus* is known as a species easily acquiring resistance genes. Therefore, most strains belonging to this species are resistant to available antimicrobial agents [6, 7].

Another CoNS that may be a threat to humans is *S. hominis*. It is described as the third most often isolated species from patients’ blood infections [8]. Mendoza-Olazaran et al. (2013) claimed that its isolates from blood commonly produced a biofilm and that they were often methicillin-resistant [9]. Pathogenicity of *S. hominis* is usually revealed with hospital-acquired bacteraemias as a result of medical procedures [10].

In the literature, *S. epidermidis* and *S. aureus* are described as the most common causes of prosthetic joint infections (PJIs), that are one of the most serious complications of prosthetic surgery associated with significant morbidity, but case studies related to *S. hominis* and *S. haemolyticus* as the main factors of this type of infections were also reported [11–13]. *S. hominis* infection can also lead to endophthalmitis associated with a capsular hypopyon [14].

It was postulated that *S. simulans* is rather an animal species, which rarely colonizes the human organism. Despite this, *S. simulans* is referred to as a cause of osteomyelitis, prosthetic joint infections as well as a habitant of the urinary tract of healthy women [15, 16]. Clinicians, especially dermatologists, should be aware that *S. simulans* can cause skin and soft tissue infections; therefore, it should not be treated as a sample contamination [17, 18]. Moreover, it is suggested that *S. simulans* is more virulent than other CoNS and leads to infections clinically similar to those caused by *S. aureus* [18, 19].

According to Torre et.al. (1992), one of the first *S. warneri* isolates came from monkey skin and nasal membranes [20]. The first isolations of this species from humans were related to implant-associated infections. It is postulated that *S. warneri* rarely causes infections in individuals that are free from risk factors such as endovascular prosthetic devices or catheters. Nevertheless, there are some case reports of *S. warneri* sepsis e.g. in immunocompetent patients with multiple abscesses [21]. It can also cause infections related to community-acquired native valve endocarditis even in patients apparently free from underlying valvular heart disease and haematogenous vertebral osteomyelitis. This species was also detected in orthopaedic cases and bacteraemia [22, 23]. It is reported that it is the next species after *S. epidermidis* that colonizes hands of nurses and neonatologists, and more than 80% of its strains were resistant to methicillin [23]. *S. warneri* is one of the predominant novobiocin-sensitive CoNS species inhabiting the skin of healthy humans [24]. Similarly to *S. epidermidis* and *S. haemolyticus*, *S. warneri* may be responsible for urinary tract infections and its presence can be associated with cellular changes in the bladder [5]. Furthermore, according to the literature, *S. epidermidis* is considered as the most common CoNS species isolated from human infections [25, 26]. As other species are rarely described as etiological factors of infections, the characteristics of virulence and resistance of these species are not completely known.

In our study, we used 80 clinical CoNS isolates of *S. haemolyticus*, *S. hominis*, *S. simulans,* and *S. warneri* species*.* The aim of our study was to detect the differences between the tested species; therefore, we examined the phenotypically demonstrated virulence factors, SCC*mec* cassette typing as well as the presence and expression of genes homologous to *S. aureus* that encoded the virulence factors. The data related to the differences among these species, their virulence factors as well as mechanisms of antibiotic resistance can lead to a better understanding of CoNS infections and thus improve the clinical prognosis and suggest an efficient treatment.

## Methods

### Tested strains

A total of 80 clinical CoNS isolates were involved in our studies: 23 *S. haemolyticus* isolates, 20 *S. hominis* isolates, 18 *S. simulans* isolates, and 19 *S. warneri* isolates. All isolates tested in the study were obtained by a diagnostic microbiology laboratory (Synevo Sp. z o.o.) of Lodz area, Poland during 2014–2016. All *S. hominis* isolates as well as six isolates of *S. haemolyticus* came from blood. Other *S. haemolyticus* and *S. simulans* isolates came mostly from infected wounds, while *S. warneri* from many different sites e.g. from the eye, the peritoneum, wounds, and the urethra.

The isolates were identified using the MALDI-TOF method as well as a genetic technique [27, 28]. *S. haemolyticus* ATCC 29970, *S. hominis subsp. hominis* ATCC 27844, *S. warn*eri ATCC 27836 and *S. simulans* ATCC 27848 were used as the control in the PCR identification tests.

### Characterisation of the virulence factors of the tested isolates

The biofilm formation was evaluated with the crystalline violet (CV) staining method after 24 h of cultivation at 37 °C in 96-well polystyrene plates under the static condition as described by Christensen et al. (1985) [29]. The ability to produce lipases was determined on a medium with the addition of triolein (ICN Biomedicals) [30] and on an agar medium supplemented with 1% of Tween 20, Tween 40, Tween 60, Tween 80 and Tween 85, respectively (ICN Biomedicals). The cultures on the Tween media were incubated for 18 h at 37 °C followed by a further 24-h incubation at a room temperature. Insoluble calcium soaps, which were visible in the form of turbidity around the growth zone, proved the enzymes activity. *S. aureus* ATCC 12600 reference strain was used as a control. The haemolytic activity of cytolysins was determined on an agar medium with 5% of sheep blood. The production of β-toxin was detected by a reverse CAMP test and an analysis of the hot–cold effect. The production of δ-toxin was detected by the CAMP test.

### The detection of phenotypic resistance to antibiotics

The sensitivity to antibiotics was determined by the disc-diffusion method (using Becton Dickinson discs) in accordance with the EUCAST guidelines. The sensitivity to the following antibiotics: cefoxitin (FOX-30), clindamycin (CC-2), erythromycin (E-15), gentamicin (GM-10), tetracycline (TE-30), ciprofloxacin (CIP-5), tigecycline (TGC-15), linezolid (LZD-30), cotrimoxazole (SXT-1.25/23.75), rifampicin (RA-5), and fusidic acid (FA-10), has been examined. *S. aureus* ATCC 29213 was used as a control strain. The results were interpreted according to the EUCAST guidelines. For LZD-30, which is not covered by EUCAST, the CLSI guidance was used.

### DNA and RNA isolation

The genomic DNA, used further for the DNA analysis, was isolated using the Genomic Micro AX Staphylococcus Gravity set (A&A Biotechnology, Poland) concordantly with the protocol provided by the manufacturer. RNA was isolated by the Total RNA Prep Plus Minicolumn Kit (A&A Biotechnology, Poland) according to the protocol provided by the manufacturer.

### An SCC*mec* analysis by PCR

The presence of the *mecA* gene was determined by PCR [31]. *S. epidermidis* ATCC 51625 was used as a control. PCR assays for typing of SCC*mec* cassettes were executed according to methods described by Kondo et al. (2007) and Zhang et al. (2005) in which specific types of the *ccr* gene complexes (*AB*1, *AB2, AB3* or *C*) and the *mec* gene complexes (*A*, *B* and *C*) were looked for [31, 32]. The *ccrAB4* gene was determined using the method proposed by Oliveira et al. (2006) with some modifications according to Zhang et al. (2013) [33, 34]. The cassettes in which the determination of the *ccr* and/or *mec* gene complex by PCR assays was not possible, were identified as “non-typable” (NT), while the cassettes that contained a previously undefined combination of these genes were classified as not yet detected (NEW) according to the principles established by the International Working Group on the Classification of Staphylococcal Cassette Chromosome Elements (2009) [35].

### The detection of the enterotoxins and haemolysin genes as well as genes encoding enzymes responsible for the PIA production (the ica operon genes)

The presence of the following genes was determined: 1. genes encoding enterotoxins: *sea* (520 bp), *seb* (643 bp), *seg* (327 bp), *sei* (465 bp); 2. genes encoding haemolysins: *hla* (209 bp), *hlb* (309 bp), *hld* (111 bp), γ-haemolysin component A (*hlgA*; 535 bp), γ-haemolysin component B (*hlgB*; 390 bp); 3. genes encoding leukocidin Panton Valentine (*pvl*; 433 bp); 4. genes encoding enzymes responsible for the PIA production - the *ica* operon (*icaA* – 103 bp, *icaD* - 198 bp, *icaB* – 302 bp, *icaC* – 400 bp, *icaR* – 469 bp genes). PCR assays for the detection of haemolysins and Panton Valentine leukocidin *pvl* genes were performed using the primers and parameters described by Jarraud et al., (2002) or Ünal and Çinar (2012), respectively [36, 37]. PCR assays for the detection of the *ica* operon genes was performed using the primers and parameters described by Arciola et al. (2005) [38]. *S. aureus* ATCC25923, and *S. epidermidis* ATCC12228 strains were used as the positive and negative controls.

### The analysis of the expression of the *pvl* and *sea* genes

The analysis of the expression of the *pvl* and *sea* genes was executed by a real-time PCR assay on the RotorGene™ 6000 thermocycler (Corbett Life Science; Qiagen). PCRs are set up using cDNA derived from the input RNA. Reverse transcriptase (RT) reaction was performed using *Enhanced Avian HS RT-PCR* Kit (Sigma) in accordance with the manufacturer’s protocol.

The PCR assay mixture for PCR amplification contained the cDNA template, 0.5 μM of each primer, 10 × AccuTaq Buffer, 0.5 U of AccuTaq LA DNA Polymerase Mix, 0.2 mM of each dNTP, and water to a final volume of 20 μl. Negative control was included in each experiment (a sample without a cDNA template). The primer sequences for both genes were planned using Primer3 software (*sea* Forward: CCTTTGGAAACGGTTAAAACG, *sea* Reverse: TCTGAACCTTCCCATCAAAAAC, 127 bp; *pvl* Forward: ATCATTAGGTAAAATGTCTGGA, *pvl* Reverse: GCATCAAGTGTATTGGATAGCAA, 179 bp).

### The statistical analysis

The associations between the results obtained for species vs cassettes type, isolation, the virulence factors prevalence were determined using the chi-squared test. *P* < 0.05 proved the significance of these relations. To analyse the differences between the tested species, the non-parametric Kruskal Wallis test was used.

## Results

The results related to the features potentially important to the colonization and invasiveness of the tested species are presented in Table 1. The biofilm formation was phenotypically observed in 84% of investigated isolates and was statistically more common in species isolated from blood (*S. haemolyticus* and *S. hominis*) than from a wound (*p*-value = 0.02).

**Table 1:** The virulence factors demonstrated phenotypically

Virulence factors			Species n/total (%)			
			*S. haemolyticus*	*S. hominis*	*S. simulans*	*S. warneri*
Biofilm formation			18/23	18/20	6/18	10/19
Lipids degradation	Triolein		23/23	20/20	0/18	19/19
	Tween	20	16/23	7/20	18/18	9/19
		40	0/23	0/20	18/18	6/19
		60	0/23	0/20	18/18	10/19
		80	0/23	4/20	18/18	14/19
		85	0/23	4/20	18/18	19/19
Haemolysis (β-type) on sheep blood medium			19/23	18/20	12/18	5/19
Haemolysins	ß-haemolysin (reverse CAMP test, hot-cold effect)		0/23	0/20	0/18	0/19
	δ-haemolysin (CAMP test)		17/23	19/20	12/18	5/19

**Fig. 1:**
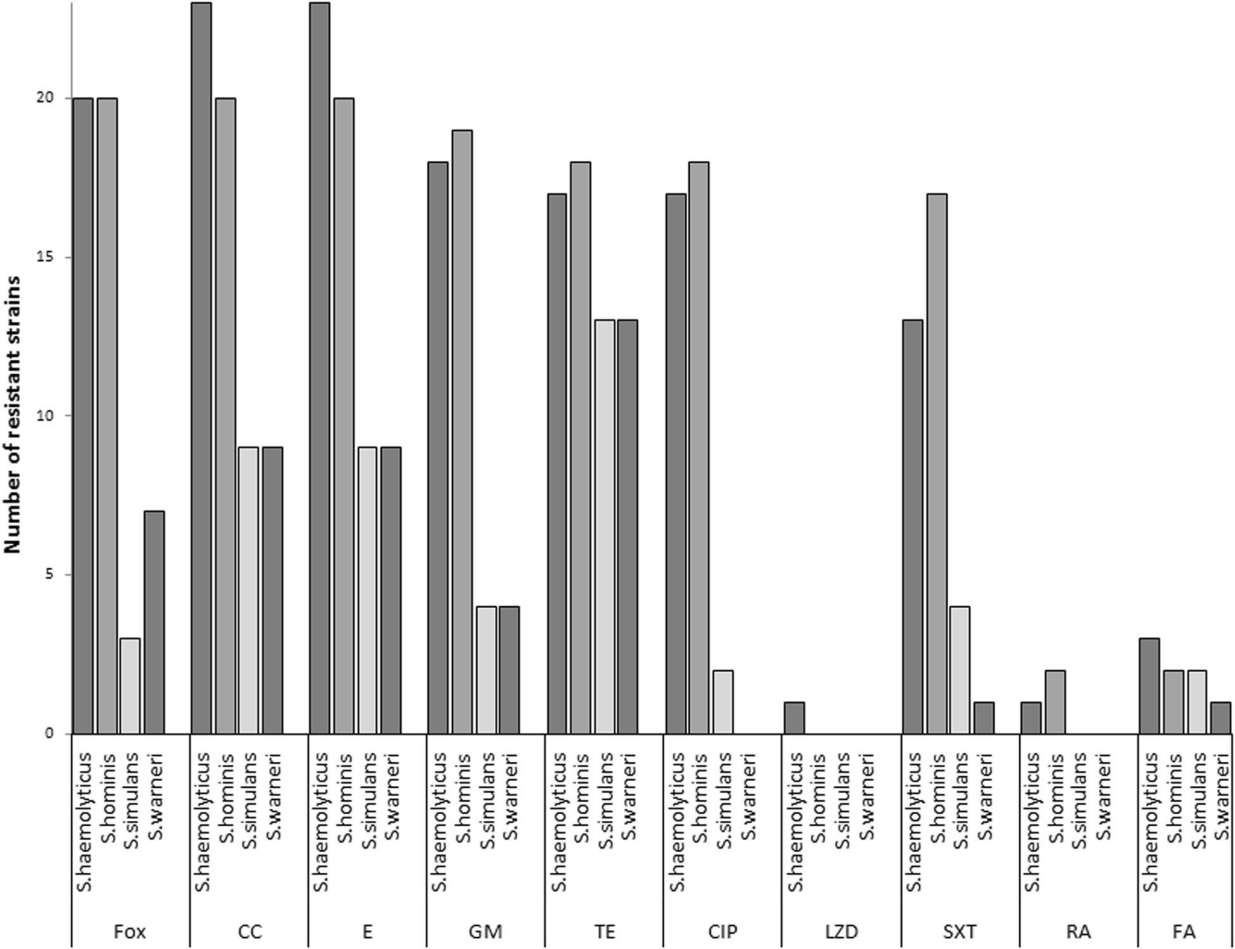
Antibiotic resistance of isolates accordingly to the species

*S. haemolyticus* and *S. hominis* isolates demonstrated a hydrolytic activity against triolein (triglyceride) but only some of them produced esterases able to release fatty acids from synthetic sorbitol esters. *S. simulans* isolates did not cause a decomposition of triolein but of hydrolysed sorbitol esters. *S. warneri* isolates presented the widest spectrum of lipolytic activity. Haemolysis on agar plates supplemented with sheep blood and a positive result of the CAMP test that indicate the ability to produce δ haemolysin, were demonstrated by 74% *S. haemolyticus* and 95% *S. hominis* isolates as well as 66% *S. simulans* isolates and 26% *S. warneri* isolates. Almost all *S. haemolyticus* and *S. hominis* isolates were clinically resistant to beta-lactams, macrolides, lincosamides (cross-resistance), aminoglycosides as well as tetracycline, ciprofloxacin, trimethoprim/sulfamethoxazole. The isolates were sensitive to tigecycline and, with the exception of one *S. haemolyticus* isolate, to linezolid (Fig. 1).

A half or a little less than a half of *S. simulans* and *S. warneri* isolates were resistant to macrolides and lincosamides (cross-resistance) as well as to tetracycline. About one third (7/19) of *S. warneri* isolates was resistant to ß-lactams while six of them were resistant to other applied antibiotics. Most of the tested isolates were sensitive to aminoglycosides and quinolones. The results presented in Fig. 1 clearly show that without a species identification, only linezolid, rifampicin, and fusidic acid can be empirically regarded as effective against the tested isolates of CoNS. The resistance to ß-lactams of all isolates with this phenotype was related to the presence of the *mecA* gene. This gene was located on the gene cassettes. The obtained results of the mec typing are presented in Table 2.

**Table 2:** Types of cassettes detected in the tested isolates

SCC*mec* cassette								Cassette type	No. of isolates
*ccr* complex					*mec* complex				
*AB1*	*AB2*	*AB3*	*AB4*	*C*	*A*	*B*	*C*		
*S. haemolyticus*									
–	–	–	–	+	–	–	+	V	6
–	–	–	–	–	–	–	+	NT	3
–	–	–	–	–	–	–	–	NT	8
–	–	–	–	+	+	–	–	NEW	2
*S. hominis*									
–	+	–	–	–	–	+	–	IV	1
–	–	–	–	+	–	–	+	VII	1
+	–	–	–	–	–	–	+	IX	2
+		–	–	+	–	–	–	NT	2
–	–	–	–	–	+	–	–	NT	6
–		–	–	–	–		+	NT	1
–	–	+	–	–	–	–	–	NT	1
–	+	–	–	–	–	–	–	NT	1
+	–	–	–	–	+	–	–	NEW	3
–	–	–	–	+	+	–	–	NEW	2
*S. simulans*									
–	–	–	–	–	+	–	–	NT	3
*S. warneri*									
–	+	–	–	–	–	+	–	IV	2
–	–	–	–	–	–	–	–	NT	4

*NT* non-typeable

The majority of the detected cassettes were non-typeable. Among 19 *S. haemolyticus* isolates with cassettes, only six were classified into class V. The presence of this cassette was statistically significant in *S. haemolyticus* isolates (*p*-value = 0.03). Typeable cassettes in *S. hominis* species belonged to type IV, VII and IX. All methicillin-resistant *S. simulans* isolates had non-typeable cassettes while *S. warneri* had type IV cassettes (two isolates) and non-typeable cassettes (four isolates).

Searching in genomes of the tested isolates for sequences homologous to genes encoding the virulence features in *S. aureus,* allowed for detection of only a few of them. The obtained results are presented in Table 3. The *hlgA* and *hlgB* genes encoding γ-haemolysin were detected only in 26% isolates. Single elements of *icaADBC* operon could not be involved in the biofilm formation.

**Table 3:** Genes detected in the tested isolates

Gene name		Species			
		*S. haemolyticus*	*S. hominis*	*S. simulans*	*S. warneri*
Haemolysin genes	*hla*	0/23	0/19	0/18	0/20
	*hlb*	0/23	1/19	0/18	0/20
	*hld*	0/23	0/19	0/18	0/20
	*hlgA*	4/23	8/19	1/18	8/20
	*hlgB*	0/23	1/19	0/18	1/20
Panton–Valentine leukocidin	*pvl*	1/23	0/19	2/18	1/20
	*pvl* expression	0/1	0/0	1/2	1/1
*icaADBC operon and icaR transcriptional repressor*	*icaA*	0/23	0/19	0/18	0/20
	*icaB*	1/23	1/19	0/18	0/20
	*icaC*	5/23	8/19	3/18	7/20
	*icaD*	1/23	0/19	0/18	0/20
	*icaR*	13/23	11/19	3/18	9/20
Enterotoxin genes	*sea*	0/23	0/19	1/18	1/20
	*sea* expression	0/0	0/0	1/1	1/1
	*seb*	0/23	0/19	0/18	0/20
	*sei*	0/23	0/19	0/18	0/20
	*seg*	0/23	0/19	0/18	0/20

One *S. haemolyticus*, one *S. warneri,* and two *S. simulans* isolates harboured the *pvl* genes. Moreover, in one *S. simulans* and one *S. warneri* isolates, the *sea* genes were found. However, the expression of *pvl* and *sea* genes was confirmed only for one *S. simulans* and one *S. warneri* isolate. None of the tested isolates exhibited an expression of both genes.

Statistically significant differences between the tested species were demonstrated in the number of genes encoding the virulence factors homologous to *S. aureus* and in the number of antibiotics that the isolates were resistant to (*p*-value = 0.04 and *p*-value = 0.00).

The highest frequency of the tested genes was observed in *S. hominis* isolates. The isolates of that species were also resistant to the highest number of antibiotics.

## Discussion

We analysed the features that are significant for the pathogenesis of four less frequently isolated CoNS species. The isolates came from the clinical material and were considered as etiological infection factors. Their relatively rare isolation provides limited data concerning the characteristic features of these species. The number of isolates analysed in our work also did not allow us to support all the obtained results by the means of a statistical analysis.

**Fig. 2:**
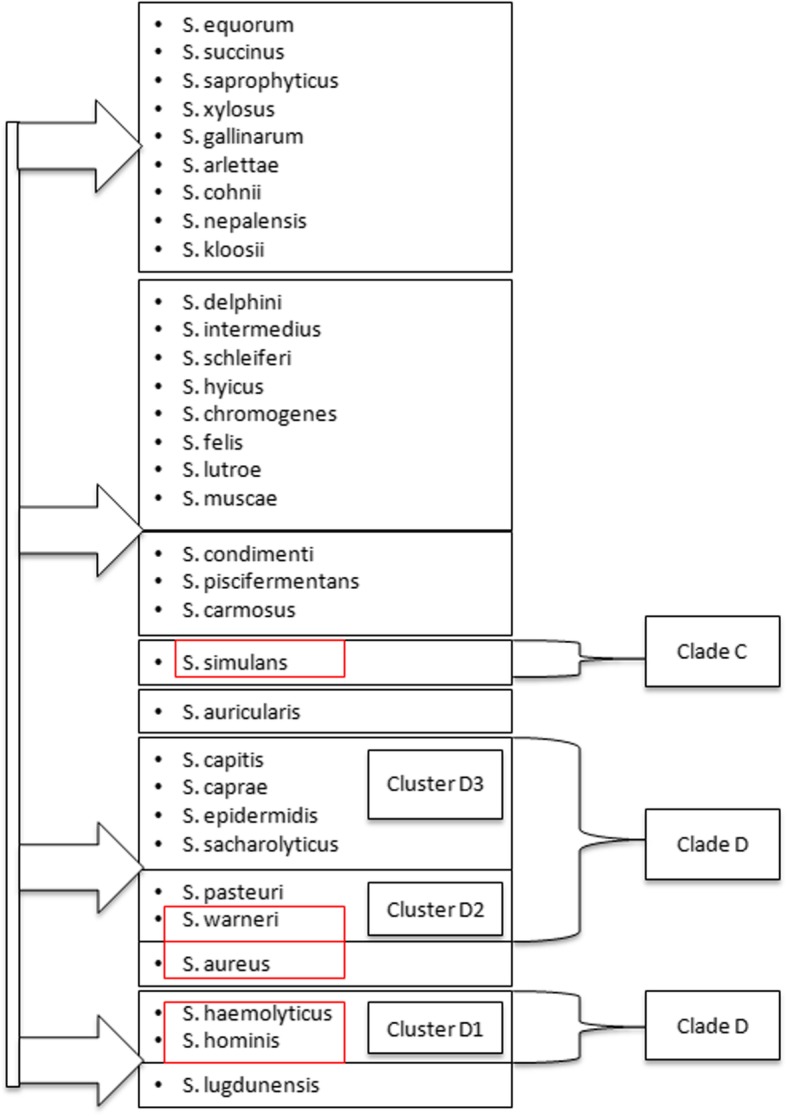
Phylogenetic tree of the species similarities of the *Staphylococcus* genus (according to de Vos et al., (2009) and Naushad et.al (2017) with own modification) [39, 40]

Staphylococci are a coherent group with phylogenetically close related species (Fig. 2). Such a close relation between the tested species as well as their common habitat may lead to a similar gene equipment or ability to acquire genes from related species by a horizontal gene transfer (HGT) process. The virulence factors in *S. aureus* are quite well known but the knowledge about the presence of virulence factors encoding genes and their expression and regulation in coagulase-negative staphylococci is not yet complete [41–43].

It is postulated that the pathogenicity of CoNS is related to their ability to form mixed biofilms [44, 45]. In our study, most *S. haemolyticus* as well as *S. hominis* isolates and significantly fewer isolates of *S. simulans* (33%) and *S. warneri* (55%) produced a biofilm. The biofilm formation by CoNS is conditioned by the presence of Microbial Surface Components Recognising Adhesive Matrix Molecules (MSCRAMMs) and the production of polysaccharide intercellular adhesin (PIA) [46, 47]. The ability to produce polymeric N-acetyl glucosamine (PNAG) is a result of the *icaADBC* operon genes activity. The *icaA* and *icaD* genes are responsible for the PIA components conformation, *icaC* is responsible for the elements involved in the transport of molecules outside the cell, while *icaB* participates in the construction of the final structure of N-acetylglucosamine polymers that are already outside the cell [48–50]. Many tested isolates contained single genes from *icaADBC* operon but none of them harboured structure genes. It suggests the presence of another *ica*-independent mechanism involved in the biofilm formation. The biofilm formation by *S. haemolyticus ica*-negative ocular isolates was also confirmed by Panda and Singh (2018) [4]. The biofilm facilitates colonization, the ability to persist in the area of the infection, HGT and antibacterial drug resistance. It is noteworthy to mention that in a bacteria-forming biofilm, the production of lipase is upregulated [51, 52]. CoNS are prevalent in areas with many sebaceous follicles [53]. They grow within the sebaceous unit superficially, where they produce extracellular enzymes with lipolytic and esterolytic activity [54]. Their activity spectrum is related to the type of infections. We observed significant differences between the tested species. *S. haemolyticus* and *S. hominis* isolates from blood demonstrated lipolytic activity mostly against triolein (lipase Geh). The hydrolysis of host lipids (e.g. triolein) leads to the release of free fatty acids, crucial for the bacterial membrane phospholipid synthesis [55]. Rollof et.al. (1987) claimed that isolates from disseminated or deep infections show stronger lipolytic activity than those from superficial infection sites [56]. Regarding the fatty acid esters, the broadest spectrum in our studies was demonstrated by *S. warneri*. The samples were isolated from different materials (wound, eye, urethra, cervix). This allows us to assume that this feature helps these species to colonize a wide spectrum of niches. Isolates able to release short-chain fatty acids were detected among all the tested species. Their concentration in certain areas might be important for the microbiome formation. Free fatty acids that are released from triglycerides, together with those already present on the skin or in a wound environment e.g. from triolein, can inhibit the growth of other bacteria in the infected sites [57]. All species described in our publication displayed differences in their abilities to hydrolyse lipids. Other authors indicated that this feature is specific to the particular species [58–60]. It might predispose the species to cause a certain type of infection. The cytolysin activity expressed by staphylococci influences their virulence [61].

In our studies, almost all *S. haemolyticus* isolates as well as *S. hominis* isolates produced haemolysis of sheep blood. It is in opposition to the literature data, according to which *S. hominis* has no haemolytic activity [39]. However, this property was not related to the presence of genes homologous to those involved in haemolytic activity of *S. aureus*. We did not find *hla* or *hlb* genes in any of the tested isolates.

The ability to produce a two-component cytolysin γ encoded by *hlgA*/*hlgC* and *hlgB* genes is assigned to the species that cause skin abscesses [62, 63]. Among the tested isolates, *S. hominis* (from blood) and *S. warneri* (from a wound) had these genes. *S. haemolyticus* (from BAL), *S. simulans* (from a wound) and *S. warneri* (from the peritoneal cavity) harboured genes involved in the synthesis of Panton-Valentine leukocidin. These genes were expressed in the isolates of *S. simulans* and *S. warneri*. The spread of new clones with these properties in a hospital environment may make them the emerging pathogens among the CoNS. PVL secreted by *S. aureus* is linked to severe life-threatening infections e.g. osteomyelitis, often accompanied by deep vein thrombosis (DVT), especially in young patients [64, 65]. PVL is mostly related to community-associated methicillin-resistant *S. aureus* (CA-MRSA) infections, particularly of skin and soft tissue and to highly lethal necrotising pneumonia [66, 67]. The *pvl* genes are located on lysogenised bacteriophages integrated into the *S. aureus* chromosome [68]. We detected also an expression of the enterotoxin gene *sea* in *S. simulans* and *S. warneri* isolates. SEA is the most common toxin causing *S. aureus*-related food poisoning [69]. The expression pattern of SEA is different from that of SEB, SEC, and SED, as it is regulated independently of the accessory gene regulator (Agr) [70]. Genes for SEA are located on the prophages, for SEB on the chromosome or plasmid, and for SEG and SEI on enterotoxin gene cluster (*egc*) and the chromosome. As most of these regions are mobile, horizontal transfer between the strains is possible [71, 72]. Among the tested isolates, we observed significant differences in their sensitivity to antibiotics. *S. haemolyticus* and *S. hominis* isolates were multi-resistant. These strains may have participated in transmission of genes related to the resistance of *S. aureus* [7, 73]. In our studies, the resistance of the tested *S. haemolyticus* isolates was equally represented by strains isolated from blood and other materials. All the *S. hominis* isolates tested by us belonged to *S. hominis* subsp. *hominis* and were resistant to all clinically important antibiotics. Up to now, another *S. hominis* subspecies, *S. hominis* subsp. *novosepticus,* was considered as multi-resistant [74]. Similarly to *S. epidermidis, S. hominis* is present on human skin more often than other CoNS [39]. They are numerous on the shoulders, legs, and the head. Together with their common multi-resistance to antibiotics and the fact that they easily cause blood infections, this accounts for an additional biohazard. Among all the tested species, *S. hominis* isolates accumulated in the most efficient way the virulence genes typical for *S. aureus*; they were also the most resistant to antibiotics. What is important, the antibiotics of choice in the treatment for such serious infections caused by MRCoNS are vancomycin and daptomycin. In the study we did not mark MIC for such antibiotics.

The resistance to the tested antibiotics was less common among *S. simulans* and *S. warneri* isolates and concerned mainly macrolides, lincosamides aminoglycosides, and tetracyclines. Detailed data related to the resistance of the tested isolates to macrolides, lincosamides, and streptogramin B were presented in our previous paper [75].

The resistance to methicillin was an important feature of the *S. haemolyticus* and *S. hominis* isolates. Our results confirmed that rifampicin and fusidic acid could be applied as an alternative treatment of infections caused by methicillin-resistant CoNS [76]. According to studies related to MRSA, 11 types of SCC*mec* cassettes have been described (International Working Group on the Classification of Staphylococcal Cassette Chromosome Elements, 2009) [35]. It is suggested that their diversity in CoNS may even be much higher [77]. In *S. haemolyticus* isolates, we detected type V cassettes, which are described in the literature as typical for this species [78, 79]. Moreover, we observed the presence of *mec* complex *C* and *ccr* complex *C* in these isolates, while their presence in other tested species was occasional. Among *S. hominis* species, *mec* complex *A* and *ccr* complex *AB1* were the most common. Such a combination of *mec* and *ccr* complexes does not correspond to any of the so far described cassettes. Other authors also confirmed the existence of this combination among the strains of *S. hominis*, also with a link to the *ccr* complex *C* [9, 77, 80]. The cassette type that was marked in our studies as ‘NEW’ and was found in *S. haemolyticus* and *S. hominis* isolates, was a new configuration containing the *mec* complex *A* with the combination of the *ccr* complex *C* or the *ccr* complex *AB1*. Data related to the presence of SCC*mec* in *S. simulans* and *S. warneri* species are sparse [81, 82]. Our results broaden this knowledge. We detected type IV cassette containing the *ccr* complex *AB2* or the *mec* complex *A* in *S. warneri* and a new combination (NEW) in other isolates.

## Conclusions

Our results highlight the increasing significance of CoNS. To the best of our knowledge, the study is the first report that indicates differences among the four tested CoNS species, less frequently isolated from human clinical specimens. The study also shows that staphylococci, mainly isolated from blood, are a serious threat for the patients. These bacteria are multi-resistant to antibiotics, which causes additional difficulties in treatment. Presented data shows that infections caused by CoNS should be a cause of concern to the clinicians. Taking into consideration that bacteremia caused by MRCoNS is treated by vancomycin and daptomycin, marking the sensitivity for these antibiotics is a target for future studies.

## Data Availability

The datasets used and/or analyzed during the current study are available from the corresponding author on reasonable request.

## Abbreviations

CC: Clindamycin
CIP: Ciprofloxacin
CoNS: Coagulase-negative staphylococci
E: Erythromycin
FA: Fusidic acid
FOX: Cefoxitin
GM: Gentamicin
LZD: Linezolid
RA: Rifampicin
SXT: Cotrimoxazole
TE: Tetracycline
TGC: Tigecycline
